# Streptococcal peritonitis in Australian peritoneal dialysis patients: predictors, treatment and outcomes in 287 cases

**DOI:** 10.1186/1471-2369-10-19

**Published:** 2009-07-26

**Authors:** Stacey O'Shea, Carmel M Hawley, Stephen P McDonald, Fiona G Brown, Johan B Rosman, Kathryn J Wiggins, Kym M Bannister, David W Johnson

**Affiliations:** 1 Australia and New Zealand Dialysis and Transplant Registry, Adelaide, Australia; 2 Department of Renal Medicine, University of Queensland at Princess Alexandra Hospital, Brisbane, Australia; 3 Department of Nephrology & Transplantation Services, University of Adelaide at the Queen Elizabeth Hospital, Adelaide, Australia; 4 Department of Nephrology, Monash Medical Center, Clayton, Victoria, Australia; 5 Renal Department, Middlemore Hospital, Otahuhu, Auckland, New Zealand; 6 University of Melbourne Department of Medicine, St Vincent's Hospital, Fitzroy, Victoria, Australia; 7 Department of Nephrology, Royal Adelaide Hospital, Adelaide, Australia

## Abstract

**Background:**

There has not been a comprehensive, multi-centre study of streptococcal peritonitis in patients on peritoneal dialysis (PD) to date.

**Methods:**

The predictors, treatment and clinical outcomes of streptococcal peritonitis were examined by binary logistic regression and multilevel, multivariate poisson regression in all Australian PD patients involving 66 centres between 2003 and 2006.

**Results:**

Two hundred and eighty-seven episodes of streptococcal peritonitis (4.6% of all peritonitis episodes) occurred in 256 individuals. Its occurrence was independently predicted by Aboriginal or Torres Strait Islander racial origin. Compared with other organisms, streptococcal peritonitis was associated with significantly lower risks of relapse (3% vs 15%), catheter removal (10% vs 23%) and permanent haemodialysis transfer (9% vs 18%), as well as a shorter duration of hospitalisation (5 vs 6 days). Overall, 249 (87%) patients were successfully treated with antibiotics without experiencing relapse, catheter removal or death. The majority of streptococcal peritonitis episodes were treated with either intraperitoneal vancomycin (most common) or first-generation cephalosporins for a median period of 13 days (interquartile range 8–18 days). Initial empiric antibiotic choice did not influence outcomes.

**Conclusion:**

Streptococcal peritonitis is a not infrequent complication of PD, which is more common in indigenous patients. When treated with either first-generation cephalosporins or vancomycin for a period of 2 weeks, streptococcal peritonitis is associated with lower risks of relapse, catheter removal and permanent haemodialysis transfer than other forms of PD-associated peritonitis.

## Background

Streptococcal peritonitis accounts for between 6% and 16% of peritoneal dialysis (PD)-associated peritonitis [1,2] and is generally reported to be severe [3]. The 2005 update of the International Society of PD (ISPD) Guidelines for Management of PD-related Infections recommends that streptococcal peritonitis is best treated with intraperitoneal ampicillin for a period of 3 weeks [3]. However, these recommendations are based on limited observations, mostly in the form of case reports or small case series [1,4-10], some of which have been potentially confounded by the inclusion of enterococci (previously called non-haemolytic streptococci and now recognised to be a separate genus of Gram-positive cocci). Moreover, there has not previously been a comprehensive examination of different therapeutic approaches to streptococcal peritonitis.

The aim of the current study was to examine the frequency, predictors, treatment and clinical outcomes of streptococcal peritonitis in all Australian PD patients involving 66 PD centres.

## Methods

### Study Population

The study included all Australian adult patients from the ANZDATA Registry who were receiving PD between 1^st ^October 2003 (when detailed peritonitis data started to be collected) and 31^st ^December 2006. Ethical approval for the use of registry data was obtained from the Princess Alexandra Hospital Human Research Ethics Committee. The data collected included demographic data, cause of primary renal disease, co-morbidities at the start of dialysis (coronary artery disease, peripheral vascular disease, cerebrovascular disease, chronic lung disease, diabetes, hypertension, and smoking status), body mass index (BMI), late referral (defined as commencement of dialysis within 3 months of referral to a nephrologist), microbiology of peritonitis episodes (up to three organisms for polymicrobial episodes) and the initial and subsequent antibiotic treatment regimens. Diagnosis of streptococcal peritonitis was made based on a PD effluent white cell count >100/μl with >50% polymorphonuclear leucocytes and isolation of *Streptococcus *species from dialysate culture. Enterococci, which are now recognised as a separate genus of Gram-positive cocci, were not included. In cases of polymicrobial peritonitis, streptococcal peritonitis was recorded if a streptococcal species was at least 1 of the isolated organisms. Centre size was categorised according to quartiles of the numbers of patients cared for by individual units over the duration of the study: small (<11 patients), small-medium (11 – 38 patients), medium-large (39 – 98 patients) and large (>99 patients).

The outcomes examined were peritonitis relapse, repeat peritonitis, peritonitis-associated hospitalization, catheter removal, temporary or permanent transfer to haemodialysis, and patient death. Peritonitis relapse was defined as an episode of peritonitis occurring within 4 weeks of the last antibiotic dose (or within 5 weeks if intermittent vancomycin used) for peritonitis due to the same organism. Repeat peritonitis was defined as an episode of peritonitis occurring more than 4 weeks after the last antibiotic dose (or more than 5 weeks if intermittent vancomycin was used) for peritonitis due to the same organism.

### Statistical analysis

Results were expressed as frequencies and percentages for categorical variables, mean ± standard deviation for continuous variables, and median and interquartile range for non-parametric data. Differences between 2 groups of patients were analysed by chi-square test for categorical data, unpaired t-test for continuous parametric data and Mann-Whitney test for continuous nonparametric data. The independent predictors of streptococcal peritonitis were determined by multivariate multilevel mixed-effects poisson regression: this hierarchical model took into account clustering based on state of residence, treating unit and individual patient [11]. Predictors of peritonitis outcomes were assessed by multivariable binary logistic regression. Data were analysed using the software packages SPSS for Windows release 12.0 (SPSS Inc., North Sydney, Australia) and Stata/SE 10.1(College Station, Tx). P values less than 0.05 were considered statistically significant.

## Results

### Population Characteristics

A total of 4675 patients received peritoneal dialysis in Australia during the study period (1 October 2003 to 31 December 2006). They were followed for 6002 patient-years. Two hundred and eighty-seven episodes of *Streptococcal *peritonitis occurred in 256 individuals. Streptococcal species accounted for 4.6% of all 3594 peritonitis episodes. The rates of all peritonitis and streptococcal peritonitis were 0.60 and 0.05 episodes per patient-year of treatment, respectively. Additional non-streptococcal organisms were isolated in 66 (23%) episodes of streptococcal peritonitis, including coagulase negative staphylococci (n = 17), *S. aureus *(n = 8), enterococci (n = 4), *Corynebacterium *(n = 1), other Gram-positive organisms (n = 2), *Pseudomonas *(n = 2), *Acinetobacter *(n = 1), *E. coli *(n = 14), *Klebsiella *(n = 5), *Serratia *(n = 1), *Proteus *(n = 1), *Enterobacter *(n = 1), *Neisseria *(n = 4), other Gram-negative organisms (n = 2), *Candida *(n = 3) and other organisms (n = 2). Fifty-seven episodes of peritonitis were associated with 2 organisms, and 9 episodes were associated with 3 organisms. Two hundred and twenty-eight patients experienced 1 episode of streptococcal peritonitis during the study period, 26 experienced 2 episodes, 1 experienced 3 episodes and 1 experienced 4 episodes.

### Predictors of *Streptococcal *peritonitis

The characteristics of patients who did and did not experience streptococcal peritonitis are shown in Table 1. On univariate analysis, patients who experienced streptococcal peritonitis during the study period were more likely to be indigenous (Aboriginal and Torres Strait Islander and Maori and Pacific Islander peoples) or Asian, be referred to a renal unit within 3 months of commencing renal replacement therapy and have a baseline PET demonstrating low average peritoneal transport status. The hierarchical multivariate poisson model showed that the only independent predictor of streptococcal peritonitis incidence was Aboriginal/Torres Strait Islander racial origin (adjusted incidence rate ratio (IRR) 1.75, 95% CI 1.008 – 3.03; p = 0.047). The development of streptococcal peritonitis was not associated with age, gender, BMI, kidney function at dialysis commencement, current smoking status, chronic lung disease, coronary artery disease, peripheral vascular disease, cerebrovascular disease, diabetes mellitus, ESRF, or late referral within 3 months of needing to start dialysis.

**Table 1:** Characteristics of all Australian PD patients who did and did not experience streptococcal peritonitis at any stage during the period 2003–2006.

Characteristic	Streptococcal peritonitis (n = 256)	No Streptococcal peritonitis (n = 4419)	p Value
Age (years)	62.4 ± 15.8	61.5 ± 16.8	0.4

Women	134 (52%)	2415 (55%)	0.5

Racial origin			0.005
Caucasian	176 (69%)	3405 (77%)	
Aboriginal/Torres Strait Islander	28 (11%)	333 (8%)	
Maori/Pacific Islander	8 (3%)	100 (2%)	
Asian	37 (14%)	404 (9%)	
Other	7 (3%)	177 (4%)	

BMI (kg/m^2^)	25.8 ± 5.1	25.9 ± 6.5	0.9

eGFR at dialysis start (mL/min/1.73 m^2^)	6.8 ± 4.3	7.1 ± 4.5	0.4

Late referral	74 (29%)	1037 (24%)	0.047

ESRF Cause			0.4
Chronic glomerulonephritis	75 (29%)	1248 (28%)	
Diabetic nephropathy	79 (31%)	1240 (28%)	
Renovascular disease	32 (13%)	607 (14%)	
Polycystic kidneys	17 (7%)	239 (5%)	
Reflux nephropathy	12 (5%)	185 (4%)	
Other	23 (9%)	622 (14%)	
Unknown	18 (7%)	278 (12%)	

Current smoker	26 (10%)	531 (12%)	0.2

Chronic lung disease	32 (13%)	567 (13%)	0.9

Coronary artery disease	99 (39%)	1568 (35%)	0.3

Peripheral vascular disease	59 (23%)	987 (22%)	0.8

Cerebrovascular disease	27 (11%)	581 (13%)	0.2

Diabetes mellitus	100 (39%)	1638 (37%)	0.5

Peritoneal Transport Status			<0.001
High	31 (12%)	440 (10%)	
High average	94 (37%)	1617 (37%)	
Low average	86 (34%)	992 (22%)	
Low	12 (5%)	186 (4%)	
Unknown/Not specified	33 (13%)	1184 (27%)	

Centre size (no. PD patients)			0.7
Small (≤ 10)	2 (1%)	53 (1%)	
Small-Med (11–38)	15 (6%)	306 (7%)	
Med-Large (39–98)	52(20%)	976 (22%)	
Large (≥ 99)	187 (73%)	3084 (70%)	

State			0.2
New South Wales	101 (39%)	1731 (39%)	
Northern Territory	5 (2%)	80 (2%)	
Queensland	52 (20%)	903 (20%)	
South Australia	20 (1%)	275 (6%)	
Tasmania	3(1%)	74 (2%)	
Victoria	40 (16%)	920 (21%)	
Western Australia	35 (14%)	436 (10%)	

### Effect of previous peritonitis episodes on occurrence of *Streptococcal *peritonitis

A history of previous peritonitis was less common for streptococcal than non-streptococcal peritonitis episodes (39% vs 45%, respectively, p < 0.05). Moreover, the time elapsed between a prior peritonitis episode and the subsequent one was significantly longer for streptococcal peritonitis (median period 140 days, interquartile range [IQR] 65.75–311.5 days) than non-streptococcal peritonitis (median 74 days, IQR 34.75–175 days, p = 0.4). Consequently, the probability of an episode of subsequent peritonitis being caused by a streptococcal species as opposed to an organism other than streptococcus significantly increased over time from 7% in the first 60 days to 6% in the first year to 11% after the first year (p < 0.05).

### Treatment of *Streptococcal *peritonitis

The vast majority of patients with *Streptococcal *peritonitis were initially treated with either intraperitoneal vancomycin or cephazolin in combination with gentamicin as empiric therapy (Table 2). Once culture results became known at a median time of 3 days, approximately one-third of patients were changed to vancomycin monotherapy (27%) or cephalothin monotherapy (8%). Oral antibiotics were prescribed at some stage during the treatment of streptococcal peritonitis in a minority of patients, the most common ones being cephalexin (11%), ciprofloxacin (8%), amoxycillin plus clavulanate (6%), amoxycillin (5%), metronidazole (3%) and flucloxacillin (3%). Overall, the median total antibiotic course duration was 13 days (Table 3). Heparin was administered to dialysate in 28 (10%) episodes of streptococcal peritonitis. Streptokinase was not instilled in the PD catheter in any episodes of streptococcal peritonitis and only 28 (10%) patients with streptococcal peritonitis received concomitant prophylactic nystatin therapy.

**Table 2:** Antimicrobial agents prescribed in initial, second and third antibiotic regimens for streptococcal peritonitis episodes in Australian PD patients 2003–2006.

Antibiotic	**1**^st ^**regimen** (n = 287)	**2**^nd ^**regimen** (n = 163)	**3**^rd ^**regimen** (n = 37)
Cephazolin	131 (46%)	24 (15%)	3 (8%)

Vancomycin	129 (45%)	77 (47%)	17 (46%)

Gentamicin	203 (71%)	20 (12%)	7 (19%)

Other Aminoglycoside	1 (0.3%)	1 (1%)	0 (0%)

Ceftazidime	21 (7%)	3 (2%)	0 (0%)

Cefoxitin	9 (3%)	1 (1%)	0 (0%)

Cefipime	1 (0.3%)	1 (1%)	0 (0%)

Cephalothin	13 (5%)	5 (3%)	0 (0%)

Ceftriaxone	10 (3%)	4 (2%)	0 (0%)

Cefotaxime	0 (0%)	2 (1%)	0 (0%)

Cephalexin	13 (5%)	14 (9%)	4 (11%)

Other cephalosporin	4 (1%)	0 (0%)	0 (0%)

Ampicillin	3 (1%)	5 (3%)	2 (5%)

Amoxycillin	1 (0.3%)	9 (6%)	3 (8%)

Amoxycillin+clavulanate	6 (2%)	8 (5%)	3 (8%)

Dicloxacillin/Flucloxacillin	4 (1%)	3 (2%)	1 (3%)

Other penicillin	2 (1%)	2 (1%)	0 (0%)

Ciprofloxacin	17 (6%)	3 (2%)	4 (11%)

Metronidazole	2 (1%)	4 (3%)	1 (2%)

Cotrimoxazole	1 (0.3%)	0 (0%)	0 (0%)

Erythromycin	0 (0%)	1 (1%)	3 (1%)

Antifungal agent	2 (1%)	5 (3%)	3 (8%)

Other	3 (1%)	6 (4%)	0 (0%)

Results represent number of episodes treated with antibiotic (% of total treated with 1^st^, 2^nd ^or 3^rd ^line regimen). Note that values within each column add to more than 100% because of the use of combination antimicrobial regimens.

**Table 3:** Treatment characteristics and clinical outcomes of PD-associated peritonitis due to streptococci or other organisms in Australia 2003–2006.

Outcome	Streptococcal peritonitis (n = 287 episodes)	Non-streptococcal peritonitis (n = 3307 episodes)	P value
Treatment			
Change to 2^nd ^antibiotic regimen	163 (57%)	1847 (56%)	0.8
Time to 2^nd ^antibiotic regimen	3 [1.75–5]	3 [2–5]	1.0
Change to 3^nd ^antibiotic regimen	37 (13%)	460 (14%)	0.7
Time to 3^rd ^antibiotic regimen	7.5 [5–9]	6 [4–10]	0.4
Total antibiotic treatment duration	13 [8–18]	14 [8–20]	0.10

Hospitalisation			
Number (%)	212 (74%)	2292 (69%)	0.11
Duration (days)	5 [3–10]	6 [3–12]	0.002

Catheter removal			
Number (%)	29 (10%)	746 (23%)	<0.001
Time to occurrence (days)	7 [3–12]	6 [3–13]	0.8

Temporary haemodialysis			
Number (%)	9 (3%)	143 (4%)	0.3
Time to occurrence (days)	3 [0.5–8]	6 [3–11.75]	0.08
Duration (days)	36 [9–89]	68.5 [24.75–104.75]	0.12

Permanent haemodialysis			
Number (%)	25 (9%)	610 (18%)	<0.001
Time to occurrence	8 [5–13]	7 [4–12]	0.4

Death			
Number (%)	4 (1%)	78 (2%)	0.3
Time to death	7 [3–99.5]	13 [3.25–23.75]	0.7

Results are expressed as number (%) or median days [interquartile range].

### Outcomes of streptococcal peritonitis

Compared with non-streptococcal peritonitis, streptococcal peritonitis was associated with significantly lower risks of relapse (3% vs 15%), catheter removal (10% vs 23%) and permanent haemodialysis transfer (9% vs 18%), as well as a shorter duration of hospitalisation (Table 3). The risk of death was lower with streptococcal peritonitis, although the difference was not statistically significant. Overall, 249 (87%) patients with streptococcal peritonitis were successfully treated with antibiotics without experiencing relapse, catheter removal or death.

The administration of vancomycin, cephalosporin or another agent in the initial or subsequent empiric antibiotic regimens did not significantly influence streptococcal peritonitis outcomes on either univariate or multivariate analyses. Longer antibiotic course duration was associated with higher risks of hospitalisation (OR 1.02 per day, 95% CI 1.008–1.02), catheter removal (OR 1.01, 95% CI 1.004–1.02) and permanent haemodialysis transfer (OR 1.01, 95% CI 1.003–1.01), presumably reflecting the medical treatment response to more severe peritonitis episodes. Using binary logistic regression, catheter removal was independently predicted by increasing age (OR 1.03 per year, 95% CI 1.0–1.07), female gender (OR 0.35, 95% CI 0.13–0.99), missing baseline PET data (OR 9.15, 95% CI 1.93–43.5), high average transport status (OR 4.12, 95% CI 1.10–15.4) and polymicrobial peritonitis (OR 7.77, 95% CI 3.00–20.1). Permanent haemodialysis transfer was independently predicted by higher BMI (OR 1.10 per kg/m^2^, 95% CI 1.01–1.28), missing baseline PET data (OR 9.78, 95% CI 2.36–40.6) and polymicrobial peritonitis (OR 9.84, 95% CI 3.71–26.1). No other variables were independently associated with either of these outcomes and there were no significant independent predictors of streptococcal peritonitis relapse, hospitalisation or death.

## Discussion

The present study, involving 287 cases of PD-associated streptococcal peritonitis across 66 different PD centres, represents the largest examination to date of the frequency, predictors, treatment and clinical outcomes of this important condition. The key findings were that streptococcal peritonitis accounted for just under 5% of all peritonitis episodes in Australia and was associated with significantly lower risks of relapse (3% vs 15%), catheter removal (10% vs 23%) and permanent haemodialysis transfer (9% vs 18%) than other forms of PD-associated peritonitis. Additional organisms were isolated in 23% of streptococcal peritonitis episodes and 21% of patients experienced more than one episode of streptococcal PD peritonitis.

These findings are in keeping with those of Shukla et al [1], who undertook a 10-year review of 104 episodes of streptococcal PD peritonitis at a single centre between 1995 and 2005. They observed that streptococcal peritonitis was associated with good outcomes with a relapse rate of 7.6%, catheter removal rate of 4.8% and median duration of hospitalisation of 8 days. Approximately one-third of patients experienced more than one episode of streptococcal peritonitis and additional organisms were isolated in 16.3% of episodes. Interestingly, Shukla and coworkers observed that streptococcal peritonitis accounted for a much higher proportion of total peritonitis episodes than in our study (11.7% vs 4.6%) and that the incidence increased from 6% in 1995–1996 to 12–16% from 1997–2005. They attributed this rise to the introduction of the Y-set system and the use of mupirocin for routine exit site care, which may have selectively suppressed staphylococcal infections resulting in an apparently higher proportion of non-staphylococcal peritonitis. In contrast, less than a third of PD units in Australia regularly use mupirocin prophylaxis, which may have accounted for part of the disparity in streptococcal peritonitis prevalences between our study and that of Shukla et al. Nevertheless, the rate of streptococcal peritonitis in our study (0.05 episodes per patient-year) was relatively low.

Previous studies have suggested that streptococcal peritonitis accounts for between 6% and 16% of all PD peritonitis [1,10,12-14]. Some of this apparent disparity in results may be accounted for by the universal use of twin-bag systems in Australia and the exclusion of enterococci, which were previously considered to be non-haemolytic strepotococci, but are now recognised as a separate genus of Gram-positive cocci [15]. For example, Munoz de Bustillo et al [10] observed 58 episodes of streptococcal PD peritonitis between 1980 and 1995, accounting for 12% of all peritonitis episodes, and reported that streptococcal peritonitis was associated with a delayed treatment repsonse. However, 19 (32.7%) of these episodes were caused by *Enterococcus faecalis*, such that true streptococcal peritonitis actually accounted for approximately 8% of total peritonitis episodes. Moreover, *E. faecalis *is known to be a more virulent organism than streptococci and therefore likely to have biased reporting of streptococcal peritonitis outcomes in an adverse direction.

A novel finding of our study was that streptococcal peritonitis was independently predicted by Aboriginal or Torres Strait Islander racial background. The influence of demographic variables such as race has not been previously studied, although streptococcal skin infections are reported to be much more frequent in Aboriginal or Torres Strait Islander peoples than the rest of the Australian population [16,17]. Socioeconomic factors, such as housing conditions, and remoteness of living are likely to be important contributors to the increased risk of streptococcal peritonitis in the present study, but could not be evaluated due to the limited data collected by the ANZDATA Registry.

In contrast to previous studies [10], we did not find an increased incidence of streptococcal peritonitis in females. This gender difference has previously been attributed to colonisation of streptococcal organisms in the female genital tract [4,6], but could have been potentially influenced by the inclusion of enterococci. This factor may also have explained the finding by Munoz de Bustillo et al [10] that streptococcal peritonitis is associated with increasing age, since enterococci are associated with bowel pathology that is found more commonly in older individuals.

Prior episodes of peritonitis have previously been reported as a risk factor for streptococcal peritonitis [10], whereas we found that a history of peritonitis was not predictive and, in fact, the time between episodes of peritonitis was longer in streptococcal cases. This observation may be related to the fact that prior antibiotic treatment for peritonitis may influence peritonitis risk in favour of other organisms, such as fungi, early on [18,19]. Others have found duration of peritoneal dialysis to be the most predictive risk factor for failure of treatment response with streptococcal peritonitis [13], which was not supported by our data. This may be due to the overall lower rates of streptococcal peritonitis noted in our study or a change in trend over time attributable to the use of disconnect systems. We did find streptococcal peritonitis was associated with lower rates of relapse, catheter removal & permanent haemodialysis transfer, which is supported by the literature to date [10,13].

Empirical antibiotic therapy in our study consisted of either intraperitoneal cephazolin or vancomycin with 70% receiving concomitant gentamicin. When streptococci are identified by microscopy or culture, the ISPD guidelines recommend intraperitoneal ampicillin and the addition of gentamicin for synergy to cover enterococcal species. Only 1% of patients in this study were initially treated with ampicillin and, once culture results were known, only 3% were subsequently treated with ampicillin. Once streptococci were identified, a majority of patients remained on a cephalosporin and one third were swapped to vancomycin. These antibiotic regimens are very similar to those reported by Shukla and coworkers [1]. The conversion of a significant minority of patients in our study to vancomycin may represent ease of administration (requiring only once weekly bag dosing), development of penicillin allergy, or *in vitro *resistance of isolated streptococci to penicillins and cephalosporins. Unfortunately, the ANZDATA Registry does not collect information on antimicrobial susceptibilities of cultured micro-organisms or the reasons for antibiotic selection.

The strengths of this study included its very large sample size and inclusiveness. We included all patients receiving PD in Australia across 66 centres during the study period, such that a variety of centres were included with varying approaches to the treatment of peritonitis. This greatly enhanced the external validity of our findings. These strengths should be balanced against the study's limitations, which included limited depth of data collection. ANZDATA does not collect important information, such as the presence of concomitant exit site and tunnel infections, patient compliance, individual unit management protocols, laboratory values (such as C-reactive protein and dialysate white cell counts), severity of comorbidities, species identification and antimicrobial susceptibilities of isolated streptococci, antimicrobial dosages or routes of antimicrobial administration. Even though we adjusted for a large number of patient characteristics, the possibility of residual confounding could not be excluded. In common with other Registries, ANZDATA is a voluntary Registry and there is no external audit of data accuracy, including the diagnosis of peritonitis. Consequently, the possibility of coding/classification bias cannot be excluded.

## Conclusion

Streptococcal peritonitis is a not infrequent complication of PD, although our analysis found a lower rate of streptococcal peritonitis compared to previous studies (4.6% vs 6–16%). The condition was more common in indigenous patients. When treated with either intraperitoneal first-generation cephalosporins or vancomycin for an average period of 2 weeks, streptococcal peritonitis was associated with lower risks of relapse, catheter removal and permanent haemodialysis transfer than other forms of PD-associated peritonitis.

## Abbreviations

ANZDATA: Australian and New Zealand Dialysis and Transplant Association Registry; BMI: Body mass index; CI: Confidence interval; eGFR; Estimated glomerular filtration rate; ESRF: End-stage renal failure; IQR: Interquartile range; IRR: Incident rate ratio; ISPD: International Society of Peritoneal Dialysis; OR: Odds ratio; PD: Peritoneal dialysis; PET: Peritoneal equilibration test.

## Competing interests

### Financial Competing interests

Professor David Johnson is a consultant for Baxter Healthcare Pty Ltd and has previously received research funds from this company. He has also received speakers' honoraria and research grants from Fresenius Medical Care. Dr Kym Bannister is a consultant for Baxter Healthcare Pty Ltd. Dr McDonald has received speaking honoraria from Fresenius Australia and Baxter Australia. The remaining authors declare that they have no financial competing interests.

### Non-Financial Competing interests

The authors declare that they have no non-financial competing interests.

## Authors' contributions

SO participated in design and co-ordination and helped to draft the manuscript. CH participated in design and co-ordination, performed the statistical analysis and read and revised the manuscript critically for important intellectual content. SP participated in design and co-ordination, acquired data, and read and revised the manuscript critically for important intellectual content. FB participated in design and co-ordination, acquired data, and read and revised the manuscript critically for important intellectual content. JR participated in design and co-ordination, acquired data, and read and revised the manuscript critically for important intellectual content. KW participated in design and co-ordination, acquired data, and read and revised the manuscript critically for important intellectual content. KB participated in design and co-ordination, acquired data, and read and revised the manuscript critically for important intellectual content. DJ was the principal investigator who conceived the study, participated in design and co-ordination, performed the statistical analysis and helped to draft the manuscript. All authors read and approved the final manuscript.

## Pre-publication history

The pre-publication history for this paper can be accessed here:

http://www.biomedcentral.com/1471-2369/10/19/prepub
